# Diagnostic accuracy of quick SOFA score and inflammatory biomarkers for predicting community-onset bacteremia

**DOI:** 10.1038/s41598-022-15408-y

**Published:** 2022-07-01

**Authors:** Takashi Matono, Maki Yoshida, Hidenobu Koga, Rie Akinaga

**Affiliations:** 1 Department of Infectious Diseases, Aso Iizuka Hospital, 3-83 Yoshio, Iizuka, Fukuoka 820-8505 Japan; 2 Department of Clinical Laboratory, Aso Iizuka Hospital, Iizuka, Fukuoka Japan; 3 Clinical Research Support Office, Aso Iizuka Hospital, Iizuka, Fukuoka Japan

**Keywords:** Diagnostic markers, Predictive markers, Infectious-disease diagnostics, Infectious diseases

## Abstract

The potential use of quick SOFA (qSOFA) score and inflammatory biomarkers as bacteremia predictors is unelucidated. Herein the aim of this study was to evaluate the diagnostic accuracy of the qSOFA score and biomarkers for predicting community-onset bacteremia. We enrolled adult outpatients with blood culture samples drawn between 2018 and 2020. Contamination, intensive care unit admission, and hemodialysis were excluded. We performed a case-control study, and analyzed 115 patients (58 with bacteremia and 57 without bacteremia). The positive likelihood ratio (LR) for bacteremia was 2.46 (95% confidence interval [CI] 0.76–9.05) for a qSOFA score ≥ 2, and 4.07 (95% CI 1.92–9.58) for tachypnea (≥ 22/min). The highest performing biomarkers were procalcitonin (area under the curve [AUC] 0.80; 95% CI 0.72–0.88), followed by presepsin (AUC 0.69; 95% CI 0.60–0.79), and C-reactive protein (AUC 0.60; 95% CI 0.49–0.70). The estimated optimal cut-off value of procalcitonin was 0.377 ng/mL, with a sensitivity of 74.1%, a specificity of 73.7%, and a positive LR of 2.82. Presepsin was 407 pg/mL, with a sensitivity of 60.3%, a specificity of 75.4%, and a positive LR of 2.46. Procalcitonin was found to be a modestly useful biomarker for predicting non-severe community-onset bacteremia. Tachypnea (≥ 22/min) itself, rather than the qSOFA score, can be a diagnostic predictor. These predictors may aid decision-making regarding the collection of blood culture samples in the emergency department and outpatient clinics.

## Introduction

The estimated incidence of sepsis is reportedly 467 per 100,000 people, with a 54% global mortality rate^1^. In high-income countries, the case-fatality rate of bloodstream infections is 12–21%^2^; thus, prompt diagnosis and proper antimicrobial treatment are warranted. Although fever often accompanies bacteremia, its accuracy for predicting bacteremia is low because fever is observed in multiple diseases/reactions, such as collagenous disease, malignancy, thrombosis, and medication^3^. Furthermore, old age (≥ 65 years) is thought to be a risk factor for bacteremia and its associated mortality; however, clinical presentation and symptoms tend to be atypical^4^. Therefore, the diagnosis of bacteremia in elderly patients is often challenging, which is a concern given the rapidly aging Japanese population.

In recent years, a bedside score termed quick SOFA (qSOFA) has been used to assess disease severity in patients with suspected septicemia^5^. Furthermore, in addition to traditional biomarkers of infection/sepsis, such as procalcitonin and C-reactive protein (CRP), a soluble CD4 subtype, presepsin, has emerged^6,7^. However, evidence of the diagnostic value of the qSOFA score and presepsin for predicting bacteremia rather than sepsis is relatively limited. Hence, the aim of this study was to evaluate the diagnostic accuracy of the qSOFA score for physical examination, including inflammatory biomarkers in laboratory evaluation, when predicting community-onset bacteremia in adults with suspected infection.

## Materials and methods

### Study design and setting

This case-control study was designed to assess the performance of qSOFA scores and biomarkers in predicting community-onset bacteremia in patients with suspected infection. This study was conducted at the Aso Iizuka Hospital (AIH), a tertiary care hospital in Fukuoka, Japan, with 1048 inpatient beds and 9392–17444 sets of blood culture tests conducted, annually. The clinical data used for this study were obtained from a laboratory database and by reviewing the medical charts at AIH.

### Study population

**Figure 1:**
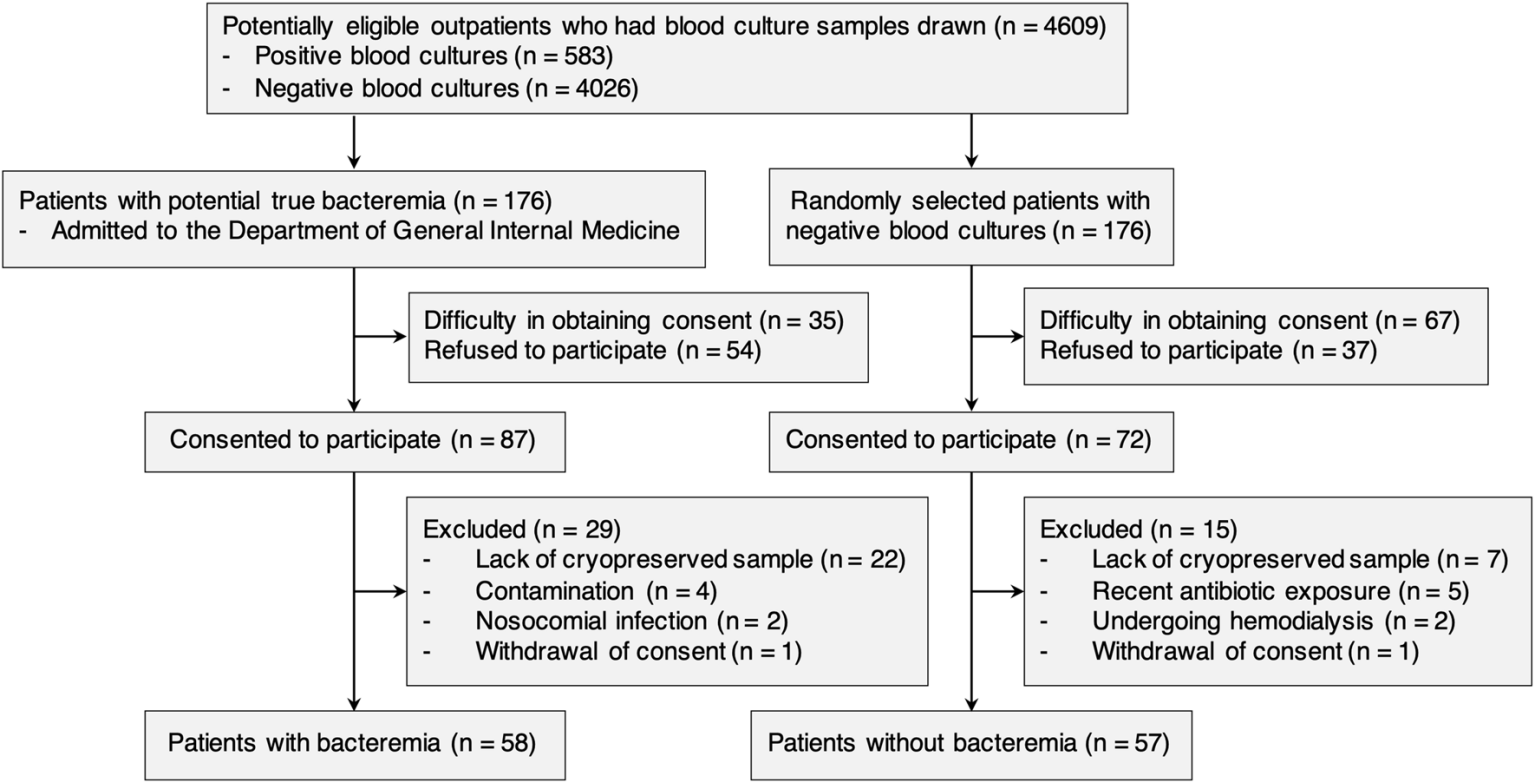
Flow diagram showing the selection criteria for patients with or without bacteremia. The reasons for difficulty in obtaining consent from patients with bacteremia included disorders of consciousness or disorientation (n = 28) and lack of written consent (n = 7). The reasons for difficulty in obtaining consent from patients without bacteremia included lack of written consent (n = 45), loss of contact (n = 16), and disorders of consciousness or disorientation (n = 6).

We included potentially eligible patients (aged ≥ 20 years) with suspected community-acquired infection who had ≥ 2 sets of blood samples for culture drawn in the emergency department and at the outpatient clinic in the Department of General Internal Medicine at AIH between September 2018 and March 2020. Among potentially eligible outpatients with positive blood cultures (n = 583), we included patients with potential true bacteremia who were admitted to the Department of General Internal Medicine (Fig. 1). Patients from whom obtaining consent was difficult (n= 35) and who refused to participate (n = 54) were excluded. We applied the exclusion criteria to the remaining 87 patients who consented to participate in the study. Consequently, 58 patients with bacteremia were included. As controls, we included randomly selected outpatients with negative blood cultures using the RAND function of Microsoft Excel, Office for Mac 2019 (Microsoft, WA, USA) in a ratio of 1 control to 1 case with potential true bacteremia (n = 176). Specifically, after confirmed culture results, we assigned a random patient number to potentially eligible controls, who matched the day of blood samples collection to the case. Following an ascending order of the random numbers, a control was randomly selected from the set of potentially eligible outpatients with negative blood cultures. After the process of control selection, 57 patients without bacteremia were included in the study. The following patients were excluded from the study: a history of recent antibiotic exposure, previously enrolled patients, admitted to the intensive care unit (ICU) (including those with severe burns and a history of resuscitation and trauma that could result in false positives for biomarkers)^6,8,9^, those undergoing renal replacement therapy (hemodialysis, chronic ambulatory peritoneal dialysis, and continuous renal replacement therapy) that could influence the presepsin value^10,11^, and patients with bacteremia identified as having contamination or nosocomial infection.

### Measurements and definitions

We extracted patient characteristics, including age, sex, underlying diseases/conditions, and site of infection, as well as vital signs and laboratory data on the day of blood culture collection. The serum of all potentially eligible outpatients who had blood samples for culture drawn was stored at the laboratory, AIH, for 2 weeks from the day of blood sample collection. CRP, procalcitonin, and presepsin at the time of blood culture collection were retrospectively measured using the cryopreserved serum samples at − 30 °C. The Charlson index was used to assess comorbidities and mortality^12^. We used the qSOFA score and Pitt bacteremia score (1998 version) to measure the acute severity of illness^5,13,14^. Creatinine clearance was predicted using the Cockcroft−Gault equation. A board-certified infectious disease physician identified true bacteremia or contamination among patients with bacteremia.

### Laboratory measurements and bacterial identification

CRP was measured by BioMajesty JCA-BM6070 (LEOL Ltd., Tokyo, Japan) using CRP-latex (II)X2 “Seiken” (Denka Seiken Co., Tokyo, Japan). Procalcitonin levels were measured by conducting an Elecsys BRAHMS PCT assay (Roche Diagnostics, Tokyo, Japan) using a Cobas e 411 analyzer (Roche Diagnostics). Presepsin levels were measured with a STACIA CLEIA Presepsin assay (LSI Medience Co., Tokyo, Japan) using the STACIA (LSI Medience Co.) system. Blood culture bottles were incubated in a BACTEC FX system (Becton, Dickinson and Co. Japan, Tokyo, Japan) for up to 7 days. Bacterial isolates recovered from blood cultures were identified by matrix-assisted laser desorption/ionization–time-of-flight mass spectrometry (MALDI–TOF MS) using a MALDI Biotyper (Bruker Daltonics, Kanagawa, Japan).

### Statistical analysis

Clinical characteristics were compared between patients with and without bacteremia. The chi-square test or Fisher’s exact test was used for nominal variables, and the Mann–Whitney U test was used for continuous variables. The diagnostic accuracy of qSOFA score ≥ 2 and each criterion for bacteremia, including sensitivity, specificity, positive/negative predictive value, or positive/negative likelihood ratio (LR), was calculated using 2 × 2 tables, along with the 95% confidence intervals (CIs). Logistic regression analysis was performed to predict potential risk factors for bacteremia based on odds ratios (ORs) and 95% CIs. The model for respiratory rate ≥ 22/min was adjusted for age, sex, body temperature, and pneumonia. Receiver operating characteristic (ROC) curve analysis was performed to derive the area under the curve (AUC), including the sensitivity, specificity, or positive/negative LR, to compare the diagnostic performance of variables in the prediction of bacteremia, and the 95% CIs were calculated. Bonferroni correction was used for multiple comparison testing of AUCs. Optimal cut-off values of the ROC curves used for predicting bacteremia were estimated using the Liu method, Youden index, and the closest-to-(0,1) method^15,16^. Statistical significance was defined as a two-tailed p-value of < 0.05, using the 95% CI. All analyses were performed using Stata/SE v. 15.1 (StataCorp, College Station, TX, USA).

### Ethical approval and consent to participate

This study was approved by the Institutional Review Board at Aso Iizuka Hospital (approval number 18036) and was conducted according to the principles of the Declaration of Helsinki. Written informed consent was obtained from the participants or their legal representatives.

## Results

### Baseline characteristics of patients

Of the 159 potentially eligible patients who consented to participate in the study (87 with bacteremia and 72 without bacteremia), 44 were excluded, and 115 were analyzed (Fig. 1). Of the 115 patients analyzed, 58 had bacteremia and 57 did not; none with neutropenia were included. Patients with bacteremia (n = 58) included 32 (55%) patients with urinary tract infection, 8 (14%) with hepatobiliary infection, 4 (7%) with intra-abdominal infection, and 4 (7%) with pneumonia (Suppl Table S1). The most common causative microorganism of bacteremia was *Escherichia coli* (28/58, 48%), followed by *Klebsiella* spp. (6/58, 10%), and *Staphylococcus aureus* (5/58, 8%; Suppl Table S2). Among the 57 patients without bacteremia, 26 (46%) had non-systemic bacterial infections, 24 (42%) had non-infectious diseases, and 7 (12%) had viral infections. Patients with a qSOFA score ≥ 2 were observed in 10/58 (17%) patients with bacteremia and 4/57 (7%) patients without bacteremia (*p* = 0.094, Table 1). Altered mental status, systolic blood pressure, and CRP levels were not significantly different between the two groups of patients studied. However, hyperthermia (median: 38.2 *vs*. 37.4 ℃, p < 0.001) and tachypnea (median: 22 *vs*. 18 /min, p <0.001) were more frequently observed in patients with bacteremia than in those without. Similarly, procalcitonin (median: 0.75 *vs*. 0.16 ng/mL, p < 0.001) and presepsin (median: 447 *vs*. 283 pg/mL, p < 0.001) were higher in patients with bacteremia than in those without.

**Table 1:** Clinical characteristics of patients with bacteremia and without bacteremia (n = 115).

Characteristics	Bacteremia (n = 58)	Non-bacteremia (n = 57)	*P* value
Age (years), median (IQR)	73 (65–85)	73 (59–83)	0.329
Male, n (%)	21 (36)	29 (51)	0.113
Days from symptom onset to visit (days), median (IQR)	1 (0–3)	2 (1–6)	< 0.001
Charlson index, median (IQR)	1 (0–2)	1 (0–3)	0.746
Pitt bacteremia score ≥ 4, n (%)	3 (5)	0 (0)	0.125
qSOFA score ≥ 2, n (%)	10 (17)	4 (7)	0.094
Altered mental status, n (%)	7 (12)	9 (16)	0.564
Body temperature (℃), median (IQR)	38.2 (37.7–39.1)	37.4 (36.7–38.0)	< 0.001
Respiratory rate (/min), median (IQR)	22 (18–24)	18 (16–20)	< 0.001
Systolic blood pressure (mmHg), median (IQR)	120 (106–140)	120 (110–135)	0.836
Total leukocytes (/μL), median (IQR)	10690 (7468–14560)	9600 (6650–12090)	0.165
Neutrophils (/μL), median (IQR)	9060 (6175–12420)	7290 (4050–9410)	0.02
C-reactive protein (mg/L), median (IQR)	62.8 (23.0–162.9)	42.0 (16.9–97.0)	0.075
Procalcitonin (ng/mL), median (IQR)	0.75 (0.35–4.25)	0.16 (0.06–0.46)	< 0.001
Presepsin (pg/mL), median (IQR)	447 (284–761)	283 (177–385)	< 0.001
Creatinine clearance* (mL/min), median (IQR)	45.8 (33.9–63.2)	57.4 (42.5–80.5)	0.027

IQR, interquartile range.

*Predicted by Cockcroft-Gault equation.

### Values of qSOFA score for predicting bacteremia

The qSOFA score ≥ 2 and each criterion (altered mental status, respiratory rate ≥ 22/min, and systolic blood pressure ≤ 100 mmHg) had low sensitivity 12.1–50% and high specificity 84.2–93% for predicting bacteremia (Table 2). The positive LR was 4.07 (95% CI 1.92–9.58) for respiratory rate ≥ 22/min and 2.46 (95% CI 0.76–9.05) for qSOFA score ≥ 2. Logistic regression analysis showed a significant association between bacteremia and respiratory rate ≥ 22/min (OR 7.14; 95% CI 2.78–18.4, p < 0.001) but did not show a qSOFA score ≥ 2 (OR 2.76; 95% CI 0.81–9.38, p = 0.10). Respiratory rate ≥ 22/min remained an independent factor for bacteremia even after adjustment (adjusted OR 6.23; 95% CI 2.16–18.0, p = 0.001).

**Table 2:** Diagnostic predictive values of each criterion regarding qSOFA score for bacteremia.

Characteristics	Sensitivity (%)	Specificity (%)	PPV (%)	NPV (%)	LR+	LR–
qSOFA score ≥ 2	17.2	93	71.4	52.5	2.46	0.89
Altered mental status	12.1	84.2	43.8	48.5	0.76	1.04
Respiratory rate ≥ 22 (/min)	50	87.7	80.6	63.3	4.07	0.57
Systolic blood pressure ≤ 100 (mmHg)	19	89.5	64.7	52	1.8	0.91

PPV, positive predictive value; NPV, negative predictive value; LR, likelihood ratio.

### ROC curve analysis on diagnostic performance

**Figure 2:**
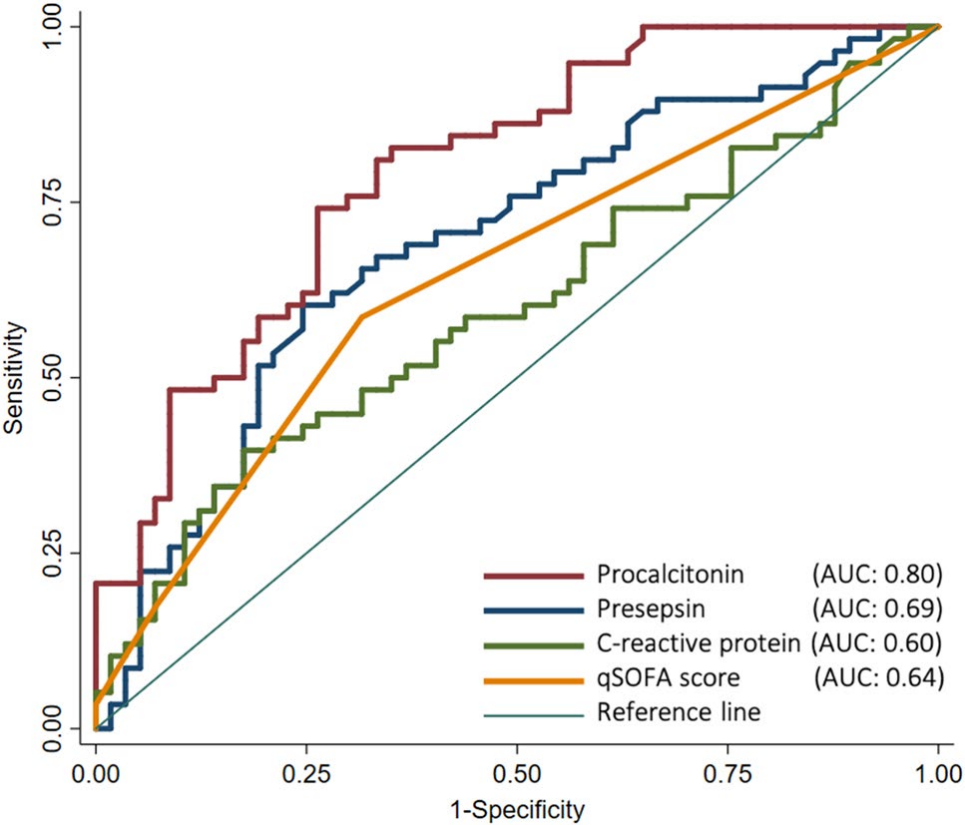
Receiver operating characteristic (ROC) curves of procalcitonin, presepsin, C-reactive protein, and qSOFA score for differentiating between patients with and without bacteremia. The ROC curve analysis showed the performance for predicting bacteremia: AUC of 0.80 (95% CI: 0.72–0.88) for procalcitonin, AUC of 0.69 (95% CI: 0.60–0.79) for presepsin, AUC of 0.60 (95% CI: 0.49–0.70) for C-reactive protein, and AUC of 0.64 (95% CI: 0.55–0.73) for qSOFA score. AUC, area under the curve; CI, confidence interval.

Among the biomarkers, ROC curve analysis revealed that the highest performance for predicting bacteremia was procalcitonin (AUC 0.80; 95% CI: 0.72–0.88), followed by presepsin (AUC 0.69; 95% CI 0.60–0.79), and CRP (AUC: 0.60; 95% CI 0.49–0.70; Fig. 2). The AUC of procalcitonin was significantly higher than that of CRP (*p* < 0 .001); however, the AUCs were not significantly different between presepsin and CRP (p = 0.50). The estimated optimal cut-off value of procalcitonin was 0.377 ng/mL, with a sensitivity, specificity, and positive LR of 74.1%, 73.7%, and 2.82, respectively, while that of presepsin was 407 pg/mL, with a sensitivity, specificity, and positive LR value of 60.3%, 75.4%, and 2.46, respectively (Table 3).

**Table 3:** Optimal cut-off values and diagnostic performance of biomarkers for predicting bacteremia.

Biomarkers	AUC	Methods	Cut-off point	Sensitivity (%)	Specificity (%)	LR+	LR–
Procalcitonin	0.80	Liu	0.38 ng/mL	74.1	73.7	2.82	0.35
		Youden					
		Closest-to-(0,1)					
Presepsin	0.69	Liu	407 pg/mL	60.3	75.4	2.46	0.53
		Youden					
		Closest-to-(0,1)					
C-reactive protein	0.60	Liu	Not detectable				
		Youden	121 mg/L	40.0	82.5	2.26	0.73
		Closest-to-(0,1)	52.2 mg/L	56.9	57.9	1.35	0.74

AUC, area under the curve; LR, likelihood ratio.

The AUC of the qSOFA score for predicting bacteremia was 0.64 (95% CI 0.55–0.73; Figure 2). The performance of body temperature and respiratory rate as predictors of bacteremia were AUCs of 0.73 (95% CI: 0.63–0.82) and 0.74 (95% CI: 0.65–0.83), respectively (Suppl Figure S1). However, leukocytosis (AUC: 0.58; 95% CI: 0.47–0.68) and neutrophilia (AUC: 0.63; 95% CI: 0.51–0.73) were low accurate predictors of bacteremia (Suppl Figure S2).

## Discussion

In this study, we assessed the diagnostic accuracy of the qSOFA score and biomarkers as predictors of community-onset bacteremia in adults with suspected infection. We found that procalcitonin (AUC: 0.80) was modestly useful as a diagnostic marker, but presepsin (AUC: 0.69) and CRP (AUC: 0.60) were not. In addition, the respiratory rate was a moderately reliable vital sign (AUC: 0.74), and tachypnea (≥ 22/min) was a substantial predictor of bacteremia (positive LR: 4.07). These findings may aid decision-making regarding the collection of blood culture samples in the emergency department and outpatient clinics.

The present study has three important findings. First, owing to the scarcity of well-designed studies that evaluated biomarkers (particularly presepsin) and their optimal cut-off values as predictors of bacteremia, our study provides valuable insights. Previous studies predominantly evaluated biomarkers as an indicator for diagnosing sepsis, rather than bacteremia. In these studies, which included systematic reviews, presepsin was shown to have a diagnostic accuracy similar to that of procalcitonin in adults^6,7^. However, an Italian study in the emergency department setting demonstrated that procalcitonin (AUC: 0.88) was superior to presepsin (AUC: 0.70) as a predictor of sepsis, which is consistent with our results^17^. Several studies have evaluated biomarkers for detecting bacteremia in adults and found that the diagnostic accuracy between presepsin and procalcitonin was similar^18–20^. However, these studies had major limitations. For instance, a Japanese study analyzing elderly patients with bacteremia in the emergency department included coagulase-negative staphylococcal contaminants in patients with bacteremia^18^. In a Spanish study, 38 of 189 (20%) patients without bacteremia had a previous history of antibiotic therapy, which might have led to false-negative results, even in a patient with true bacteremia^19^. In another Italian study, 6 of the 92 (6.5%) patients included were admitted to ICU, but the study lacked information on past medical history (such as end-stage renal diseases) and a history of trauma and burn injuries, which might have led to false positive results in the biomarkers^20^. Therefore, we believe that our study design and population, particularly our exclusion criteria, reduced the confounding factors with respect to biomarker evaluation, which allowed us to obtain more reliable findings than those in previous reports^18–20^.

Second, to the best of our knowledge, this is one of the few studies to indicate that the respiratory rate may be a significant predictor for non-severe community-onset bacteremia, particularly in elderly patients. Falguera et al. suggested that among patients with bacteremia caused by community-acquired pneumonia, tachypnea (>30/min) was one of the predictors of bacteremia^21^. However, many previous studies failed to reveal an association between tachypnea (> 20/min) and bacteremia among febrile adults in the emergency department setting^22–24^. Chou et al. described tachypnea (> 20/min) to be modestly related to bacteremia in younger adults (aged < 65 years), but not in elderly adults (aged ≥ 65 years) in the emergency department. Therefore, it is noteworthy that our findings suggest that tachypnea (≥ 22/min) can be a substantial predictor of community-onset bacteremia, even in our study population involving mostly elderly patients. Conversely, previous studies have shown that the qSOFA score was a poor predictor of bacteremia (AUC: 0.60), which is also similar to our findings^25,26^. In the emergency department setting, Furuta et al. demonstrated that the sensitivity and specificity of qSOFA-positive scores (≥ 2) for predicting bacteremia were 47.0% and 61.8%, respectively^27^. Thus, in emergency department and outpatient clinic settings, we believe that tachypnea itself, rather than the qSOFA score, is a key physical examination to predict non-severe bacteremia, even in elderly patients, as in our study population.

Third, this study sheds light on the importance of clinical reasoning in diagnosing bacteremia, instead of basing it on a laboratory result or biomarker alone. Our study suggests that leukocytosis, neutrophilia, and elevation of CRP are no longer accurate predictors of bacteremia. These findings are consistent with previous reports that showed the AUCs for total leucocyte count, neutrophil count, and CRP were 0.53–0.60, 0.57–0.63, and 0.60, respectively^19,20,28,29^. However, normal food consumption (> 80% consumed, negative LR: 0.18) and Shapiro’s clinical prediction rule (negative LR: 0.08) were thought to decrease the probability of bacteremia^3,30,31^. Conversely, shaking chills (positive LR: 4.7) and low premorbid performance status (*i.e*., bedridden and need attendance, positive LR: 3.6) reportedly increase the probability of bacteremia^3,32^. Furthermore, Pfitzenmeyer et al. suggested that a clinician’s impression of a high probability of bacteremia (≥ 50%) increases the likelihood of a positive LR of 2.3^33^. Hence, the previous reports and our findings suggest that in addition to classical laboratory markers, patient history and physical examination are also vital in the prediction of community-onset bacteremia.

The present study has several limitations. First, this was a single-center study with a small sample size, and potential selection bias may have existed, particularly in patients with bacteremia. For example, some patients with disorders of consciousness or disorientation (28 with bacteremia and 6 without bacteremia), which could relate to the qSOFA score result, were not included in the study because of the difficulty of gaining consent. Thus, it is uncertain whether our findings can be generalized to other populations. However, this study appropriately dealt with the potential confounding factors regarding the biomarker evaluation provided above. Second, we excluded ICU admission, considering the confounders; thus, our findings cannot be applied to patients with severe bacteremia. Nevertheless, our study design revealed that tachypnea (≥ 22/min) can be a key predictor for non-severe bacteremia, which is useful in the emergency department and outpatient clinic settings. Third, the estimated optimal cut-off values of biomarkers in this study had limited information regarding predicting bacteremia because each biomarker did not have an excellent diagnostic accuracy. Therefore, clinicians should determine clinically-relevant sensitivity/specificity by reference to Supplemental Table S3–S9. Forth, based on our research question, we studied the qSOFA score and biomarkers. However, we did not examine patient history, other physical examinations, and laboratory data used to predict bacteremia, such as neutrophil fraction and bacteremia-specific clinical prediction rules^23,31,34^. Therefore, further studies are required to elucidate the impact of these factors in predicting bacteremia.

In conclusion, the present findings showed that procalcitonin, rather than presepsin and CRP, was a modestly useful marker for non-severe bacteremia, and that tachypnea (≥ 22/min) itself, rather than the qSOFA score, can be used as a key bacteremia predictor. Therefore, we believe that these predictors will be useful in making decisions about the collection of blood culture samples in the emergency department and outpatient clinics.

## Supplementary Information

Supplementary Information 1.

Supplementary Information 2.

Supplementary Information 3.

Supplementary Information 4.

## Data Availability

The dataset analyzed in this study is available from the corresponding author upon reasonable request.
