# Supplementary material for: Diagnostic accuracy of quick SOFA score and inflammatory biomarkers for predicting community-onset bacteremia
Source: Sci Rep. 2022 Jul 1;12:11121. doi: 10.1038/s41598-022-15408-y (PMC9249749; doi:10.1038/s41598-022-15408-y)
Supplement: Supplementary file 1 — Supplementary Information 1. [file 41598_2022_15408_MOESM1_ESM.docx]

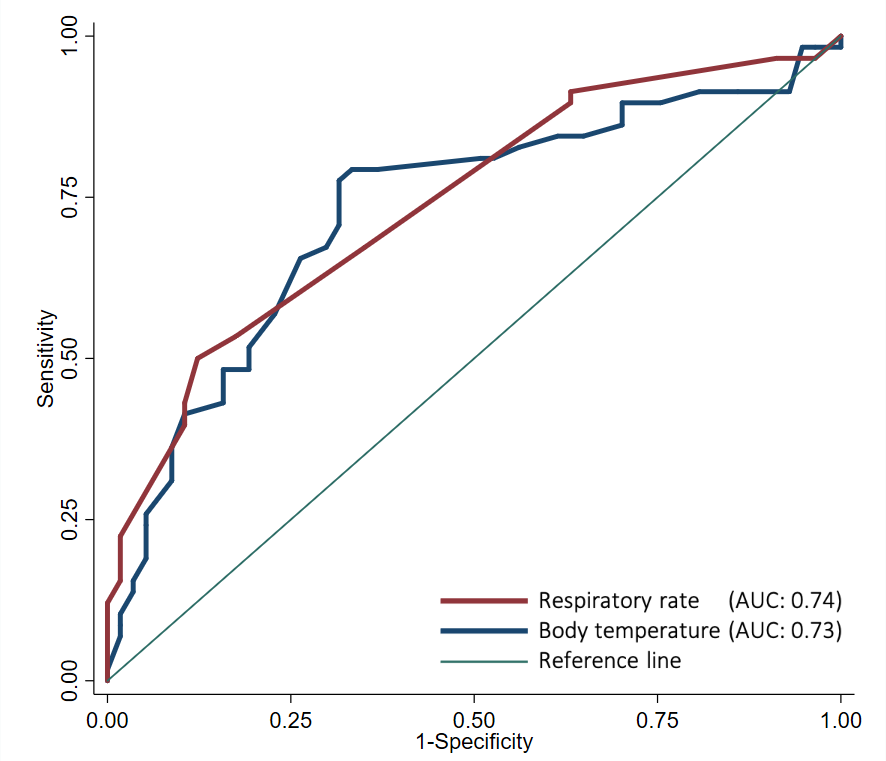


**Figure S1**. Receiver operating characteristic curves of body temperature and respiratory rate among patients with and without bacteremia.
