# Supplementary material for: Diagnostic accuracy of quick SOFA score and inflammatory biomarkers for predicting community-onset bacteremia
Source: Sci Rep. 2022 Jul 1;12:11121. doi: 10.1038/s41598-022-15408-y (PMC9249749; doi:10.1038/s41598-022-15408-y)
Supplement: Supplementary file 3 — Supplementary Information 3. [file 41598_2022_15408_MOESM3_ESM.docx]

| Table S1. Site of infection in patients with bacteremia (n = 58) | |
| --- | --- |
| Type of infection | n (%) |
| Urinary tract infection | 32 (55) |
| Hepatobiliary infection | 8 (14) |
| Intra-abdominal infection | 4 (7) |
| Pneumonia | 4 (7) |
| Skin and soft tissue infection | 3 (5) |
| Bone and joint infection | 2 (3) |
| CRBSI | 2 (3) |
| Odontogenic infection | 2 (3) |
| Infective endocarditis | 1 (2) |
| CRBSI, catheter related blood stream infection | |

| Table S2. Causative microorganism in patients with bacteremia (n = 58) | |
| --- | --- |
| Microbe | n (%) |
| Gram negative organism (n = 38) | |
| *Escherichia coli* | 28 (48) |
| *Klebsiella* spp.^*^ | 6 (10) |
| Other Enterobacterales | 3 (5) |
| *Pseudomonas aeruginosa* | 1 (2) |
| Gram positive organism (n = 15) | |
| *Staphylococcus aureus* | 5 (8) |
| Other *Staphylococcus* spp. | 2 (3) |
| Viridans streptococci | 4 (7) |
| *Aerococcus urinae* | 2 (3) |
| *Streptococcus pneumoniae* | 1 (2) |
| *Streptococcus agalactiae* | 1 (2) |
| Polymicrobial (n = 5) |  |
| * Included four *K. pneumoniae* and two *K. variicola* strains | |
